# Characterization of the complete mitochondrial genome of *Ergatettix serrifemora* (Orthoptera: Tetrigidae) from China and its phylogenetic analysis

**DOI:** 10.1080/23802359.2020.1773346

**Published:** 2020-06-05

**Authors:** Xiao-Dong Li, Xiao-Li Ying, Wei-An Deng, Rong-Jiao Zhang, Ran Li

**Affiliations:** a School of Chemistry and Bioengineering, Hechi University, Yizhou, PR China; b The Key Laboratory of Jiangsu Biodiversity and Biotechnology, College of Life Sciences, Nanjing Normal University, Nanjing, PR China

**Keywords:** *Ergatettix serrifemora*, Orthoptera, Tetrigidae, mitogenome, phylogenetic analysis

## Abstract

The mitochondrial genome (mitogenome) of the *Ergatettix serrifemora* (Orthoptera: Tetrigidae: Tetriginae) was sequenced and annotated. The assembled mitochondrial genome was 14,947 bp, containing 45.8% of A, 15.7% of C, 9.6% of G and 28.9% of T, respectively, which is the classical structure for insect mitogenome. The region that we failed to sequence was between rrnS and trnI, and generally contained a putative AT-rich region. Twelve PCGs started with typical ATN codon and eleven ended with complete stop codons (three with TAG, eight with TAA). The phylogenetic trees in the current study confirmed that *E. serrifemora* was clustered with other Tetriginae species, and this study would improve our understanding for the mitogenomes of Tetrigoidea.

The genus *Ergatettix* belongs to the subfamily Tetriginae, within the family Tetrigidae of the order Orthoptera. This genus currently includes 19 known species, which are mainly distributed in India, Nepal, Myanmar, Philippines, Malaysia, New Guinea, Iran and China (Deng 2016). Up to now, no mitochondrial sequence has been reported of the genus. In this study, the partial mitochondrial genome (mitogenome) of *E. serrifemora* (GenBank accession No. MN938923) was sequenced and determined using next-generation sequencing method for the first time, which will facilitate future studies on species identification, population genetics, and phylogenetic analysis of the subfamily Tetriginae.

Total genomic DNA was extracted from legs of adult specimen of *E. serrifemora* which was collected from Shanghang county in Fujian province, China, in August 2019 and voucher specimen are deposited in the Museum of Insects of Hechi University, label number is No. O203. The genomic DNA was sequenced using the Hiseq2500 platform (Illumina Inc., San Diego, CA). The mitogenome was assembled using Geneious (Kearse et al. 2012), version 10.2.3 (http://www.geneious.com/). In addition, all genes were annotated with MITOS Web Server (http://mitos.bioinf.uni-leipzig.de/index.py) (Bernt et al. 2013) and tRNA scan-SE server (Lowe and Chan 2016).

The mitogenome of *E. serrifemora* was 14,947 bp, containing 22 transfer RNA genes (tRNAs), 13 protein-coding genes (PCGs), 2 ribosomal RNA genes (rrnL and rrnS). The region that we failed to sequence was between *rrnS* and *trnI*, and generally contained a putative AT-rich region. The composition of the genome contained 45.8% A, 15.7% C, 9.6% G, and 28.9% T, showing an obvious A + T bias (74.7%). Nine PCGs and 14 tRNA genes were transcribed from the majority strand, while the remaining four PCGs (ND1, ND4, ND4L, and ND5), eight tRNAs and two rRNAs were located on the minority strand. In addition, the gene composition and order were similar to all reported tetrigid species. Twelve PCGs started with typical ATN codon (seven with ATG, two with ATT, one with ATG and one with ATA), whereas the ND6 gene appeared to start with TTG. Eleven PCGs ended with complete stop codons (three with TAG, eight with TAA), and COIII and ND5 ended with the incomplete stop codons T (TA), which were presumably completed as TAA by post transcriptional polyadenylation (Anderson et al. 1981).

The phylogenetic relationships of *E. serrifemora* were reconstructed using Bayesian Inference (BI) by MrBayes 3.1.2 (Huelsenbeck and Ronquist 2001) and Maximum Likelihood (ML) by RAxML 8.2.0 (Stamatakis 2014), based on 13 PCGs (10,950 bp) from mitogenomes of night tetrigid species and one outgroup (*Mirhipipteryx andensis*), respectively. Two phylogenetic trees using different methods yielded the same topology, and nodal supporting values were always higher for BI tree than for ML tree (Figure 1). The phylogenetic tree confirmed that *E. serrifemora* was closely related to other five species from Tetriginae, and all the species of the subfamily Tetriginae formed a clade. Presently, the studies recorded for Tetrigoidea was limited and we hope that our data can be useful for further study.

**Figure 1.**
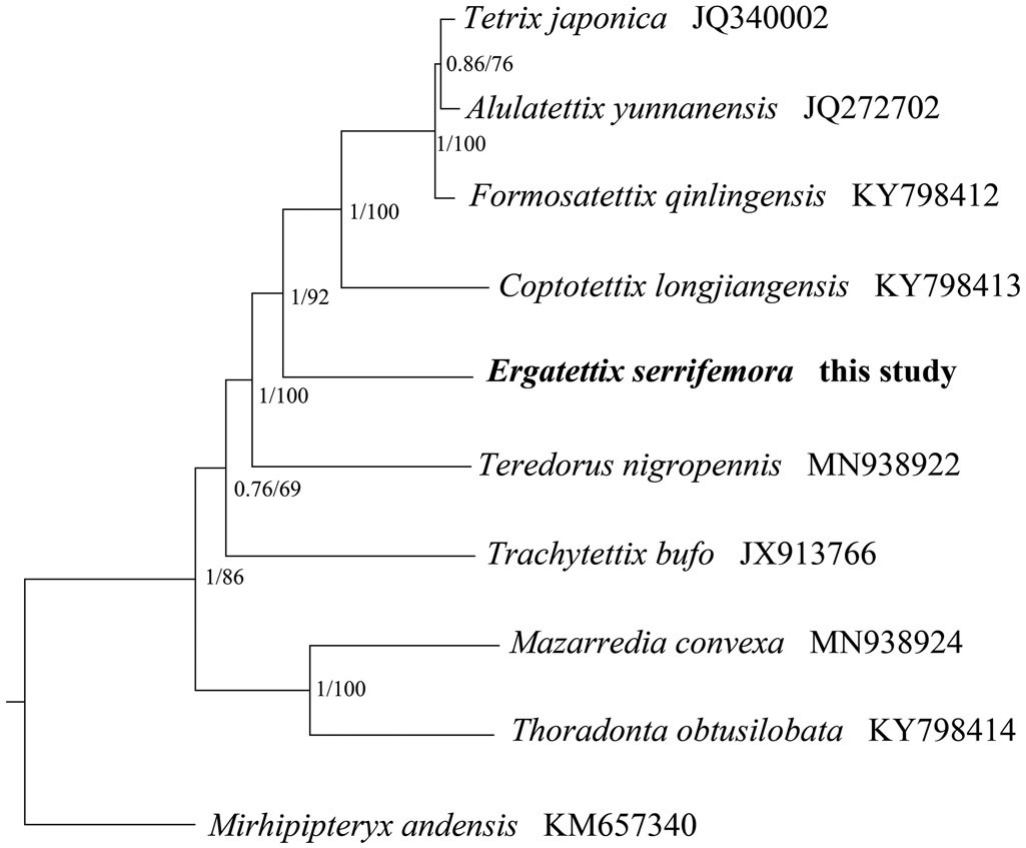
Phylogenetic tree obtained from ML and BI analysis based on 13 concatenated mitochondrial PCGs. Numbers on node are posterior probability (PP) and bootstrap value (BV).

## Data Availability

The data that support the findings of this study are openly available in [National Center for Biotechnology Information] at [https://www.ncbi.nlm.nih.gov/nuccore], reference number [MN938923].
